# Durability of single-dose HPV vaccination in young Kenyan women: randomized controlled trial 3-year results

**DOI:** 10.1038/s41591-023-02658-0

**Published:** 2023-12-04

**Authors:** Ruanne V. Barnabas, Elizabeth R. Brown, Maricianah A. Onono, Elizabeth A. Bukusi, Betty Njoroge, Rachel L. Winer, Denise A. Galloway, Leeya F. Pinder, Deborah Donnell, Imelda N. Wakhungu, Charlene Biwott, Syovata Kimanthi, Kate B. Heller, Diane G. Kanjilal, Daniel Pacella, Susan Morrison, Elena A. Rechkina, Stephen L. Cherne, Torin T. Schaafsma, R. Scott McClelland, Connie Celum, Jared M. Baeten, Nelly R. Mugo, the KEN SHE Study Team, Peter Dull, Reena Gulati, Sara Vernam, Abdul Rawuf Yousufzay, Krissa Gunderson, Amra Hercinovic, Lisa Ondrejcek, Gavin Robertson, Angela Williams, Elizabeth Brown, Jody Carter, Denise Galloway, Leeya Pinder, Priya R. Prabhu, Robin Smith, Marci Wright, Stephen O. Abiero, Maqline A. Achola, Meldah O. Adipo, Katherine L. Amukonyi, Cynthia Akinyi, Teresia O. Akinyi, Penina N. Amboka, Karl D. Arum, Veronica O. Atogo, Pius O. Atonga, Adero J. Cate, Daisy Chepkoros, Oyamo O. Christopher, Imelda N. Imali, Mildred Imbayi, Lizzie N. Kabete, Enericah K. Kanampiu, Geoffrey Kebaso, Dennis Kegode, Timothy Kwena, Reina Lenturkana, Celestine Lihavi, David N. Marwa, Patricia Matti, Peter O. Mboya, Elijah Mbuya, Lyna A. Memo, Robai M. Mituyi, Benard M. Muga, David E. Muhoma, Elizabeth L. Musi, Gilbert C. Mutai, Simon M. Muthusi, Ivy M. Mutuiri, Catherine W. Mwakio, Bill Nyongesa, Maureen A. Ochieng, Vincent R. Ochuka, Belder A. Odedo, Esther A. Odeny, Vincent O. Odera, Debora A. Odhiambo, Perez O. Odhiambo, Janet A. Okeyo, Linet A. Okode, Nollyne A. Okuku, Irene Okumu, Lydiah A. Okumu, Christine A. Olweny, Hellen A. Olweyo, George O. Omondi, Donnavane A. Ondego, Florence A. Ondiek, Joan A. Ongere, Maricianah Onono, Kevin O. Onyango, Annette A. Opondo, Millicent A. Oronje, Beryl A. Osoga, Rebecca A. Otieno, Job A. Ouma, Mildred A. Owenga, Samya S. Rashid, Vincent K. Salano, Moses O. Siaji, Roseline Sikolia, Imelda N. Wakhungu, Nicholas Walukana, Nobert B. Walusala, Caren A. Wemali, Faith Ambiyo, Emily Anyango, Esther K. Charles, David Chege, Jane Gacheru, Anne Gaitho, Stephen Gakuo, Zachary Gathu, Mathew Irungu, Vincent Juma, Irene Kamau, Mary Kibatha, Dorcas Kiboi, Francis Khaemba, Hellen W. Kimani, Catherine Kiptinness, Caren Koli, Erick Koome, Solomon Maina, Linet Makena, Sarah Mbaire, Veronica Muchoki, Victor Munene, Edwin Mugo, Nelly R. Mugo, Umi W. Mugo, Faith Munyaka, Paul Mutunga, Margaret Mwangi, Stanley Mwangi, Peter Mwenda, Gladys Namboka, Grace Ndung’u, Rispa Nduuru, Esther Neema, Sammy Ng’ang’a, Josephine Njeri, Irene Njeru, Alice Njoki, John Njoroge, Sarah Njoroge, Peter Nzuve, Fridah Nkatha, Jemimah Nyakio, Edna Nyandiga, Jacinta Nyokabi, Rose Odera, John Okumu, Lynda Oluoch, Linda Orwa, Nina Ouko, Matilda Saina, Agata Thumi, Innes Wambui, Victoria Wambui, Virginia Wangechi, Scholastica Wanjiku, Ruanne Barnabas, Francesca Caramazza, Kate Heller, Diane Kanjilal, Kyle Kennedy, Odunayo Kolawole Talabi, Rukiya Hassan, Emmanuel Kabare, Fatma H. Mwidadi, Khamis Mwinyikai, Salwa Mustafa, Juma Shafi, Stephen L. Cherne, Daphne Hamilton, Rachel Johnson, John Lin, Justice Quame-Amaglo, Elena A. Rechkina, Torin T. Schaafsma

**Affiliations:** 1 Division of Infectious Diseases, Department of Medicine, Massachusetts General Hospital, Boston, MA USA; 2 School of Medicine, Harvard Medical School, Boston, MA USA; 3 Department of Epidemiology, T. H. Chan Harvard School of Public Health, Boston, MA USA; 4 Vaccine and Infectious Disease Division, Fred Hutchinson Cancer Center, Seattle, WA USA; 5 Public Health Sciences, Fred Hutchinson Cancer Center, Seattle, WA USA; 6 Department of Biostatistics, University of Washington, Seattle, WA USA; 7 Center for Microbiology Research, Kenya Medical Research Institute, Kisumu, Kenya; 8 Department of Global Health, University of Washington, Seattle, WA USA; 9 Department of Obstetrics and Gynecology, University of Washington, Seattle, WA USA; 10 Center for Clinical Research, Kenya Medical Research Institute, Nairobi, Kenya; 11 Department of Epidemiology, University of Washington, Seattle, WA USA; 12 Human Biology Division, Fred Hutchinson Cancer Center, Seattle, WA USA; 13 University of Cincinnati, Department of Obstetrics and Gynecology, Cincinnati, OH USA; 14 Department of Laboratory Medicine and Department of Pathology, University of Washington, Seattle, WA USA; 15 Division of Allergy and Infectious Diseases, Department of Medicine, University of Washington, Seattle, WA USA; 16 East Africa STI Laboratory, University of Washington, Mombasa, Kenya; 17 Bill and Melinda Gates Foundation, Seattle, WA USA; 18 DF/Net Research, Seattle, WA USA

**Keywords:** Translational research, Preventive medicine

## Abstract

Cervical cancer burden is high where prophylactic vaccination and screening coverage are low. We demonstrated in a multicenter randomized, double-blind, controlled trial that single-dose human papillomavirus (HPV) vaccination had high vaccine efficacy (VE) against persistent infection at 18 months in Kenyan women. Here, we report findings of this trial through 3 years of follow-up. Overall, 2,275 healthy women aged 15–20 years were recruited and randomly assigned to receive bivalent (*n* = 760), nonavalent (*n* = 758) or control (*n* = 757) vaccine. The primary outcome was incident-persistent vaccine type-specific cervical HPV infection. The primary evaluation was superiority analysis in the modified intention-to-treat (mITT) HPV 16/18 and HPV 16/18/31/33/45/52/58 cohorts. The trial met its prespecified end points of vaccine type-specific persistent HPV infection. A total of 75 incident-persistent infections were detected in the HPV 16/18 mITT cohort: 2 in the bivalent group, 1 in the nonavalent group and 72 in the control group. Nonavalent VE was 98.8% (95% CI 91.3–99.8%, *P* < 0.0001) and bivalent VE was 97.5% (95% CI 90.0–99.4%, *P* < 0.0001). Overall, 89 persistent infections were detected in the HPV 16/18/31/33/45/52/58 mITT cohort: 5 in the nonavalent group and 84 in the control group; nonavalent VE was 95.5% (95% CI 89.0–98.2%, *P* < 0.0001). There were no vaccine-related severe adverse events. Three years after vaccination, single-dose HPV vaccination was highly efficacious, safe and conferred durable protection. ClinicalTrials.gov no. NCT03675256.

## Abstract (web summary)

In a prespecified 3-year analysis of the KEN SHE trial, single-dose HPV vaccination was shown to be well tolerated and provided durable protection against cervical HPV infection in Kenyan women aged 15–20 years.

## Main

Cervical cancer burden remains high globally, with more than 600 000 cases and 340 000 deaths in 2020 and incidence and mortality rates in most countries higher than the World Health Organization (WHO) threshold for cervical cancer elimination^1^. Further, there are notable disparities; cervical cancer incidence is three times higher and mortality is six times higher in countries with a United Nations Development Programme-defined low Human Development Index (HDI) than in countries with a very high HDI. Targeted strategies are needed to achieve the WHO goal of cervical cancer elimination and to reduce global cervical cancer disparities.

HPV vaccines prevent more than 90% of persistent oncogenic vaccine type-specific HPV infections, the primary cause of cervical cancer^2,3^. HPV vaccination is foundational in the WHO’s Global Cervical Cancer Elimination Strategy as a primary prevention of HPV infection^4^. The strategy aims to vaccinate 90% of girls globally. Four HPV vaccines are licensed to be given as 2–3 intramuscular injections over 2–6 months, all targeting high-risk (oncogenic) HPV types that cause 70–90% of cancers. The bivalent vaccines (Cervarix and Cecolin) prevent HPV 16/18 infection, the quadrivalent vaccine (Gardasil) prevents HPV 16/18/6/11, including the low-risk HPV types 6 and 11 to prevent genital warts, and the nonavalent vaccine (Gardasil-9) prevents HPV 16/18/31/33/45/52/58/6/11 infection, including five additional high-risk HPV types. Vaccinating the current global cohort of women aged 9–18 years would prevent HPV-associated precancerous lesions^5^ and 11.6 million cases of cervical cancer over their lifetimes^6^; however, current HPV vaccine coverage remains low. In 2019, only 15% of adolescent girls globally were vaccinated against HPV^7^.

Single-dose HPV vaccination would simplify the logistics and reduce costs of scaling up vaccine programs, lowering barriers to reaching high HPV vaccine coverage. The vaccine virus-like-particle (VLP) structure, which self-assembles to mimic the live virus without the replicating DNA, generates strong immunity with a single dose, analogous to highly immunogenic whole-virus vaccines rather than a subunit vaccine, supporting a biological mechanism for single-dose efficacy rather than the prime-boost multi-dose schedule that optimizes subunit vaccine efficacy (VE)^8^. Single-dose HPV vaccine efficacy is comparable to the licensed two- or three-dose regimen in randomized trials and observational studies^9–12^. Thus, in April 2022, the WHO recommended one or two doses of HPV vaccines for children, adolescents and young adults aged 9–20 years; however, a desire for data about longer-term durability of single-dose HPV vaccination persists^13–15^ and national guidelines continue to recommend multi-dose strategies. Also, few low-HDI countries have catch-up vaccination programs for persons 15 years and older, although those programs accelerate the impact of vaccination^5^.

In Kenya, the age-standardized incidence for cervical cancer is 31.3 per 100,000 person-years; annually, an estimated 5,236 new cases are diagnosed and 3,211 deaths attributable to cervical cancer occur^1^. Kenya’s two-dose HPV immunization program was launched in October 2019 to reach 10-year-old girls, in the context of vaccine supply constraints. In 2021, vaccine coverage for the first dose was 77% and 31% for the second dose^16^. With WHO guidance recommending vaccination for the multi-age cohort of 9–14 year olds, the easing of vaccine supply constraints, and the need to deliver immunization services to a larger number of adolescents, evaluating the efficacy of single-dose vaccination would provide evidence to policymakers for immunization scale-up, including multi-age cohorts and catch-up vaccination for those who may have missed vaccination during programmatic scale-up.

This study evaluated zero versus single-dose HPV vaccination and employed a superiority design to support efficacious, feasible, and timely evidence for catch-up vaccination^17,18^. As reported previously, at 18 months, bivalent and nonavalent vaccine efficacy was 97.5% for HPV 16/18 and nonavalent VE was 88.9% for HPV 16/18/31/33/45/52/58^10^. We hypothesized that single-dose HPV VE would be durable over 36 months. Here we report the final single-dose HPV VE 3 years after vaccine administration to evaluate the durability of single-dose HPV vaccination for zero versus single-dose HPV vaccination. As planned, all participants have received HPV vaccination and follow-up continues to evaluate the durability of single-dose efficacy.

## Results

### Participant disposition and characteristics

**Fig. 1:**
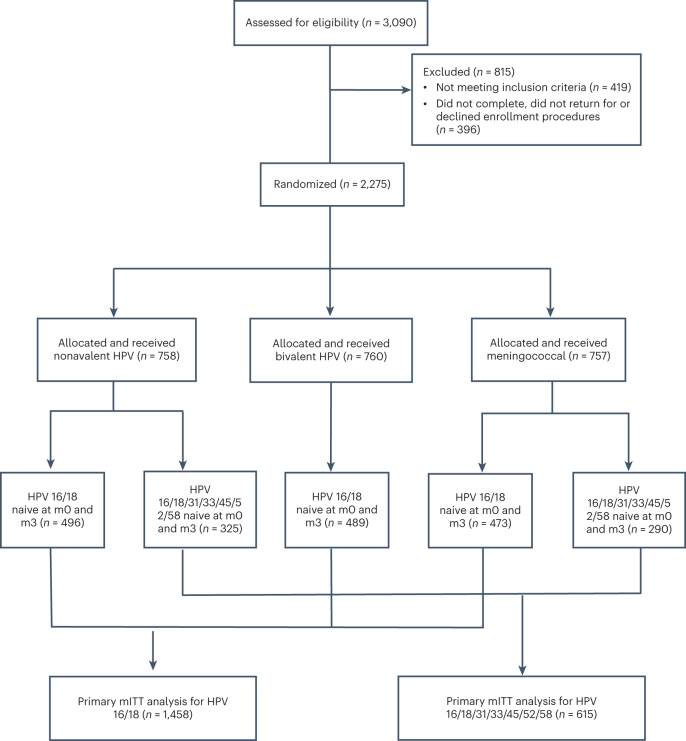
Randomized trial profile. CONSORT diagram for the disposition of KEN SHE Study participants, including primary mITT cohort disposition for HPV 16/18 and HPV 16/18/31/33/45/52/58.

Between 20 December 2018 and 15 November 2019, 3,090 participants were screened for study eligibility and 2,275 (74%) were enrolled. Of those ineligible (*n* = 419), 132 (32%) had a positive pregnancy test, 51 (12%) declined study procedures, 34 (8%) had a positive rapid HIV test and 202 (48%) met other exclusion criteria. Enrolled participants were randomized (Fig. 1): 758 to the nonavalent HPV vaccine group, 760 to the bivalent HPV vaccine group and 757 to the control vaccine group. At enrollment, 57% of participants (*n* = 1,301) were aged 15 to 17 years and 61% (*n* = 1,392) had one sexual partner in their lifetime with comparable baseline characteristics between the groups (Extended Data Table 1).

Participants included in the primary analysis tested HPV DNA negative (external genital/lateral vaginal and cervical swabs) at enrollment, by self-collected vaginal swab at month 3, and HPV antibody negative at enrollment in the mITT cohort. For inclusion in the HPV 16/18 mITT cohort, participants were HPV 16/18 naive. Similarly, for the HPV 16/18/31/33/45/52/58 mITT cohort, participants were HPV 16/18/31/33/45/52/58 naive.

For HPV 16/18, participants who tested HPV 16/18 antibody-positive or HPV 16/18 DNA-positive at enrollment or HPV DNA-positive month 3 (*n* = 661) or had missing antibody results (*n* = 1) or a missing month 3 swab (*n* = 155) were excluded. Among the 1,458 participants meeting the criteria for the primary HPV 16/18 mITT analysis, 496 were in the nonavalent group, 489 were in the bivalent group and 473 were in the control group. For HPV 16/18/31/33/45/52/58, participants who tested HPV 16/18/31/33/45/52/58 antibody- or HPV 16/18/31/33/45/52/58 DNA-positive at enrollment or HPV DNA-positive at month 3 (*n* = 792) or had missing antibody results (*n* = 1) or a missing month 3 swab (*n* = 107) were excluded. Of the 615 participants eligible for the primary HPV 16/18/31/33/45/52/58 analysis, 325 were in the nonavalent group and 290 were in the control vaccine group. The median age was 17 years for the HPV 16/18 and HPV 16/18/31/33/45/52/58 mITT cohorts (Table 1) and, overall, the baseline characteristics by study groups were comparable.

**Table 1:** Baseline characteristics of the mITT cohorts

		HPV 16/18 mITT			HPV 16/18/31/33/45/52/58 mITT	
		Nonavalent HPV	Bivalent HPV	Control	Nonavalent HPV	Control
Characteristic	Category					
	Total	496	489	473	325	290
Age group (years)	15–17	299 (60.3%)	278 (56.9%)	278 (58.8%)	197 (60.6%)	168 (57.9%)
	18–20	197 (39.7%)	211 (43.1%)	195 (41.2%)	128 (39.4%)	122 (42.1%)
Age (years)	Median (IQR)	17 (16, 18)	17 (16, 19)	17 (16, 19)	17 (16, 18)	17 (16, 19)
Marital status	Never married	478 (96.4%)	462 (94.5%)	446 (94.3%)	315 (96.9%)	269 (92.8%)
	Married	14 (2.8%)	24 (4.9%)	20 (4.2%)	7 (2.2%)	15 (5.2%)
	Previously married	3 (0.6%)	3 (0.6%)	7 (1.5%)	2 (0.6%)	6 (2.1%)
	Other	1 (0.2%)	0 (0.0%)	0 (0.0%)	1 (0.3%)	0 (0.0%)
Education (highest level)	No schooling	1 (0.2%)	2 (0.4%)	1 (0.2%)	1 (0.3%)	1 (0.3%)
	Primary school, some or complete	40 (8.1%)	30 (6.1%)	36 (7.6%)	27 (8.3%)	27 (9.3%)
	Secondary school, some or complete	359 (72.4%)	368 (75.3%)	355 (75.1%)	241 (74.2%)	220 (75.9%)
	Post-secondary school	96 (19.4%)	89 (18.2%)	81 (17.1%)	56 (17.2%)	42 (14.5%)
Earns an income of her own	No	437 (88.1%)	417 (85.3%)	417 (88.2%)	284 (87.4%)	248 (85.5%)
	Yes	59 (11.9%)	72 (14.7%)	56 (11.8%)	41 (12.6%)	42 (14.5%)
Has a current main or steady sexual partner	No	144 (29.0%)	152 (31.1%)	145 (30.7%)	98 (30.2%)	95 (32.8%)
	Yes	352 (71.0%)	337 (68.9%)	328 (69.3%)	227 (69.8%)	195 (67.2%)
Age when first had vaginal intercourse (years)	<15	123 (24.8%)	116 (23.7%)	103 (21.8%)	80 (24.6%)	65 (22.4%)
	15–17	265 (53.4%)	274 (56.0%)	282 (59.6%)	185 (56.9%)	173 (59.7%)
	≥18	96 (19.4%)	93 (19.0%)	79 (16.7%)	54 (16.6%)	46 (15.9%)
	Do not remember	12 (2.4%)	6 (1.2%)	9 (1.9%)	6 (1.8%)	6 (2.1%)
Lifetime number of sex partners	1	322 (64.9%)	332 (67.9%)	289 (61.1%)	217 (66.8%)	184 (63.4%)
	2	121 (24.4%)	100 (20.4%)	113 (23.9%)	78 (24.0%)	65 (22.4%)
	≥3	53 (10.7%)	57 (11.7%)	71 (15.0%)	30 (9.2%)	41 (14.1%)
Condom use with last vaginal sex	No	153 (30.8%)	155 (31.7%)	140 (29.6%)	98 (30.2%)	78 (26.9%)
	Yes	237 (47.8%)	235 (48.1%)	238 (50.3%)	156 (48.0%)	144 (49.7%)
	No sex in past year	106 (21.4%)	99 (20.2%)	95 (20.1%)	71 (21.8%)	68 (23.4%)
Syphilis	Negative	496 (100.0%)	489 (100.0%)	471 (99.6%)	325 (100.0%)	289 (99.7%)
	Positive	0	0	1 (0.2%)	0	1 (0.3%)
	Not done	0	0	1 (0.2%)	0	0
*Chlamydia* *trachomatis*	Negative	438 (88.3%)	434 (88.8%)	413 (87.3%)	293 (90.2%)	252 (86.9%)
	Positive	58 (11.7%)	55 (11.2%)	60 (12.7%)	32 (9.8%)	38 (13.1%)
*Neisseria* *gonorrhoeae*	Negative	488 (98.4%)	480 (98.2%)	466 (98.5%)	322 (99.1%)	285 (98.3%)
	Positive	8 (1.6%)	9 (1.8%)	7 (1.5%)	3 (0.9%)	5 (1.7%)
HSV-2	Negative	407 (82.1%)	387 (79.1%)	375 (79.3%)	264 (81.2%)	226 (77.9%)
	Positive	88 (17.7%)	102 (20.9%)	98 (20.7%)	60 (18.5%)	64 (22.1%)
	Indeterminate	1 (0.2%)	0	0	1 (0.3%)	0
BV^a^	Negative	415 (83.7%)	378 (77.3%)	378 (79.9%)	278 (85.5%)	239 (82.4%)
	Positive	81 (16.3%)	111 (22.7%)	95 (20.1%)	47 (14.5%)	51 (17.6%)
*Trichomonas* *vaginalis*	Negative	477 (96.2%)	468 (95.7%)	452 (95.6%)	315 (96.9%)	275 (94.8%)
	Positive	19 (3.8%)	21 (4.3%)	21 (4.4%)	10 (3.1%)	15 (5.2%)

^a^Nugent scores 7–10 were classified as BV positive and Nugent scores 0–6 were classified as BV negative. BV, bacterial vaginosis; IQR, interquartile range; HSV, herpes simplex virus.

One hundred percent of participants received their assigned vaccine and no administration errors were identified. Overall, 19 of 2,275 (0.8%) participants did not contribute follow-up time after enrollment and 5 (0.2%) exited the study during follow-up. Overall, 2,256 of 2,275 (99%) participants contributed a median of 35 months of follow-up time between December 2018 and January 2023. A total of 34% of participants (771 of 2,256) provided a final analysis swab at month 30 and 62% (1,397 of 2,256) at month 36 as participants received cross-over vaccination at their next study visit after regulatory approvals were obtained to allow timely access to the effective intervention. Retention of four or more swabs collected at follow-up for the assessment of primary end points was 96% (2,182 of 2,275) and 91% (2,061 of 2,2,275) for five or more swabs (Extended Data Table 2). Of the end-point swabs, 93% of swabs were cervical and 7% of swabs were self-collected vaginal swabs, which was similar across intention-to-treat (ITT) and mITT cohorts (Extended Data Table 3).

The incidence of persistent non-vaccine HPV types (HPV 26/35/39/40/42/43/44/51/53/54/56/59/61/66/68/69/70/73/82) was comparable between the three study groups: 24.9 of 100 woman-years in the nonavalent group, 25.8 of 100 woman-years in the bivalent group and 22.0 of 100 woman-years in the control group (Extended Data Table 4). The rates of chlamydia and gonorrhea were comparable across the three study groups (Extended Data Table 5).

### Primary outcomes

**Fig. 2:**
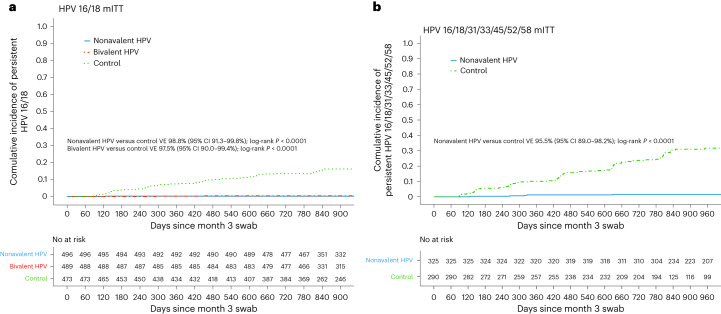
Cumulative incidence curves for the incidence of persistent HPV in the modified intention-to-treat primary analyses. Cumulative incidence curves were computed by vaccine group using Kaplan–Meier methods. Two-sided log-rank *P* values were computed for each comparison using the log-rank test. **a**, Cumulative incidence of persistent HPV 16/18 in the HPV 16/18 mITT cohort (*n* = 1,458). Four participants in the HPV 16/18 mITT cohort did not contribute a second end-point swab and thus did not contribute time at risk. **b**, Cumulative incidence of persistent HPV 16/18/31/33/45/52/58 in the HPV 16/18/31/33/45/52/58 mITT cohort (*n* = 615). One participant in the HPV 16/18/31/33/45/52/58 mITT cohort did not contribute a second end-point swab and thus did not contribute time at risk.

Through month 36, a total of 75 incident-persistent infections were detected in the HPV 16/18 mITT cohort: 1 among the nonavalent vaccine group, 2 among participants assigned to the bivalent vaccine group, and 72 among those assigned to the control vaccine group (Table 2a) (thus, no additional infections in the nonavalent group, one additional infection in the bivalent group and 36 additional infections in the control group compared to month 18). Through month 36, the incidence of persistent HPV 16/18 was 0.08 per 100 woman-years in the nonavalent vaccine group and 0.16 per 100 woman-years in the bivalent group, compared to 6.70 per 100 woman-years in the control vaccine control group. Nonavalent VE was 98.8% (95% CI 91.3–99.8%, *P* < 0.0001) and bivalent VE was 97.5% (95% CI 90.0–99.4%, *P* < 0.0001) (Fig. 2a).

**Table 2:** Incidence of persistent HPV and vaccine efficacy

a										
	HPV 16/18									
	Nonavalent HPV		Bivalent HPV		Control		Nonvalent versus control		Bivalent versus control	
	Events/ participants	Incidence of persistent HPV 16/18 per 100 woman-years (95% CI)	Events/ participants	Incidence of persistent HPV 16/18 per 100 woman-years (95% CI)	Events/ participants	Incidence of persistent HPV 16/18 per 100 woman-years (95% CI)	VE (95% CI)	*P* value	VE (95% CI)	*P* value
mITT Primary	1/496	0.08 (0–0.44)	2/489	0.16 (0.02–0.58)	72/473	6.70 (5.24–8.44)	98.8% (91.3–99.8%)	<0.0001	97.5% (90.0–99.4%)	<0.0001
mITT sensitivity	1/569	0.07 (0–0.39)	3/561	0.21 (0.04–0.62)	84/543	6.87 (5.48–8.51)	99.0% (92.5–99.9%)	<0.0001	96.8% (90.0–99.0%)	<0.0001
Extended sensitivity	0/429	0 (0–0.38)	0/404	0 (0–0.40)	44/380	5.52 (4.01–7.42)	100.0%* (NC)	<0.0001	100.0%* (NC)	<0.0001

b						
	HPV 16/18/31/33/45/52/58					
	Nonavalent HPV		Control		Nonvalent versus control	
	Events/ participants	Incidence of persistent HPV 16/18/31/33/45/52/58 per 100 woman-years (95% CI)	Events/ participants	Incidence of persistent HPV 16/18/31/33/45/52/58 per 100 woman-years (95% CI)	VE (95% CI)	*P* value
mITT primary	5/325	0.61 (0.20–1.42)	84/290	13.8 (11.0–17.0)	95.5% (89.0–98.2%)	<0.0001
mITT sensitivity	8/437	0.74 (0.32–1.45)	116/392	14.4 (11.9–17.2)	94.8% (89.3–97.4%)	<0.0001
Extended sensitivity	1/264	0.17 (0–0.92)	50/210	12.1 (8.97–15.9)	98.6% (90.0–99.8%)	<0.0001

NC, not calculated. *VE computed as 100 × (1 − crude incidence rate ratio).

Incidence of persistent HPV by randomized vaccine group in the mITT primary, mITT sensitivity cohorts. For the HPV types specified, the mITT primary cohort includes participants who were HPV DNA and antibody negative at enrollment and DNA negative at month 3; the mITT sensitivity cohort includes participants who were HPV DNA negative at enrollment and month 3; and the extended-sensitivity cohort includes participants who were HPV DNA and antibody negative at enrollment, and DNA negative and months 3 and 6. Woman-years of follow-up time is computed from the month 3 swab collection date for the mITT primary and sensitivity cohorts, and from the month 6 swab collection date in the extended-sensitivity cohort. No multiplicity adjustments were performed. **a**, Incidence of persistent HPV 16/18 and VE. For the extended-sensitivity cohort comparisons, VE is reported as 100 × (1 − crude incidence rate ratio) due to 0 events in the nonavalent and bivalent HPV vaccine arms. Two-sided log-rank *P* values are computed for each comparison using the log-rank test. **b**, Incidence of persistent HPV 16/18/31/33/45/52/58 and VE. Two-sided log-rank *P* values are computed for each comparison using the log-rank test.

At month 18, there were 33 incident-persistent infections in the HPV 16/18/31/33/45/52/58 mITT cohort: 4 in the nonavalent group and 29 in the control group. Through month 36, 89 incident-persistent infections were detected in the HPV 16/18/31/33/45/52/58 mITT cohort: 5 in the nonavalent vaccine group and 84 in the control vaccine group (Table 2b) (thus, 1 additional infection in the nonavalent group and 55 additional infections in the control group). Through month 36, the incidence of persistent HPV 16/18/31/33/45/52/58 was 0.61 per 100 woman-years in the nonavalent vaccine group compared to 13.8 per 100 woman-years in the control group. Nonavalent VE for HPV 16/18/31/33/45/52/58 was 95.5% (95% CI 89.0–98.2%, *P* < 0.0001) (Fig. 2b).

### Secondary outcomes and efficacy analyses

In the planned secondary sensitivity analysis, including participants with type-specific HPV antibodies detected at enrollment, there were 88 incident-persistent infections in the HPV 16/18 mITT cohort: 1 in the nonavalent vaccine group, 3 among participants assigned to the bivalent group and 84 among those assigned to the control vaccine group (Table 2a). HPV 16/18 incidence was 0.07 per 100 women-years in the nonavalent group, 0.21 per 100 women-years in the bivalent vaccine group and 6.87 per 100 women-years in the control group; nonavalent VE was 99.0% (95% CI 92.5–99.9%, *P* < 0.0001) and bivalent VE was 96.8% (95% CI 90.0–99.0%, *P* < 0.0001; Table 2a). In the sensitivity analysis, there were a total of 124 incident-persistent infections in the HPV 16/18/31/33/45/52/58 mITT cohort: 8 among participants assigned to the nonavalent group and 116 among those assigned to the control group; nonavalent VE was 94.8% (95% CI 89.3–97.4%, *P* < 0.0001; Table 2b).

In the planned secondary extended-sensitivity analysis, excluding participants with HPV DNA detected at month 6, there were a total of 44 incident-persistent infections in the HPV 16/18 mITT cohort: 0 each among participants assigned to the bivalent and nonavalent vaccine groups and 44 among those assigned to the control vaccine group (Table 2a). HPV 16/18 incidence was 0 per 100 women-years in the nonavalent and bivalent vaccine groups and 5.52 per 100 women-years in the control group; nonavalent VE was 100% (*P* < 0.0001) and bivalent VE was 100% (*P* < 0.0001) (Table 2a). In the extended-sensitivity analysis, there were a total of 51 incident-persistent infections in the HPV 16/18/31/33/45/52/58 mITT cohort: 1 among participants assigned to the nonavalent group and 50 among those assigned to the control group; nonavalent VE was 98.6% (95% CI 90.0–99.8%, *P* < 0.0001) (Table 2b).

In the planned secondary analyses to assess VE in the prespecified subgroups, as defined at enrollment, for the presence of co-infections (chlamydia, gonorrhea, herpes simplex type 2, trichomoniasis, syphilis and bacterial vaginosis), self-reported condom use, number of self-reported lifetime sex partners (1 versus 2+) and contraceptive method use, there was no difference in VE in predefined subgroups (Extended Data Tables 6 and 7).

### Safety

Serious adverse events (SAEs) were experienced by 201 participants, which included 122 participants with pregnancy-related SAEs, 71 with infections or inflammatory conditions (of which 39 were malaria), 7 injuries and 12 mental health illnesses. Overall, the SAE frequency was similar between groups (Table 3). There were five deaths in the study due to unsafe abortion, sepsis, suicide, hepatocellular carcinoma, complications following an emergency cesarean section for fetal distress and one unknown cause with acute symptoms of cough productive of bloody sputum. SAEs were assessed as not related to the study vaccines. Five participants had abnormal cervical cytology at enrollment and were followed until the lesions resolved or the participant received treatment. Social harms were reported by 0.31% of participants (*n* = 7), including partner physical and verbal abuse and lack of social support from friends and family for trial participation.

**Table 3:** Participants experiencing adverse events (ITT)

	Randomized group			
	Nonavalent HPV	Bivalent HPV	Control	All
Enrolled, *n*	758	760	757	2,275
Any SAE, *n* (%)	59 (7.8%)	72 (9.5%)	70 (9.2%)	201 (8.8%)
Any pregnancy-related, *n* (%)	44 (5.8%)	45 (5.9%)	33 (4.4%)	122 (5.4%)
Any infection/inflammation, *n* (%)	13 (1.7%)	26 (3.4%)	32 (4.2%)	71 (3.1%)
Any injury, *n* (%)	0 (0%)	3 (0.4%)	4 (0.5%)	7 (0.3%)
Any mental health, *n* (%)	3 (0.4%)	4 (0.5%)	5 (0.7%)	12 (0.5%)

Participants may have more than one event across categories.

### Exploratory analyses

In exploratory analysis to evaluate cross-protection against related HPV types, bivalent VE against incident-persistent HPV 31/33/45 was 10.1% (95% CI −38.7% to 41.7%) (Extended Data Table 8).

### Post hoc analyses

Using only provider-collected end-point cervical swabs and excluding self-collected vaginal swabs, the results for the primary analysis were not different: the VE was 98.7% (95% CI 90.5–99.8%) for the nonavalent vaccine and 97.3% (95% CI 89.0–99.3%) for the bivalent in the HPV 16/18 mITT cohort. Nonavalent VE was 95.3% (95% CI 88.4–98.1%) in the HPV 16/18/31/33/45/52/58 mITT cohort (Table 4).

**Table 4:** Incidence of persistent HPV and vaccine efficacy using cervical swabs only (mITT primary cohorts)

						95% CI		Statistical comparisons			
Randomized group	Enrolled (*n*)	HPV type naive at baseline (mITT) (*n*)	Incident-persistent HPV (*n*)	Woman-years of follow-up	Incidence of persistent HPV per 100 woman-years	Lower bound	Upper bound	Comparison	VE	95% CI	*P* value (log-rank)
**HPV 16/18 mITT cohort**											
Nonavalent HPV	758	496	1	1,240.76	0.08	0	0.45	Nonavalent HPV versus control	98.7%	(90.5–99.8%)	<0.0001
Bivalent HPV	760	489	2	1,216.42	0.16	0.02	0.59	Bivalent HPV versus control	97.3%	(89.0–99.3%)	<0.0001
Control	757	473	66	1,057.86	6.24	4.83	7.94				
**HPV 16/18/31/33/45/52/58 mITT cohort**											
Nonavalent HPV	758	325	5	813.66	0.61	0.20	1.43	Nonavalent HPV versus control	95.3%	(88.3–98.1%)	<0.0001
Control	757	290	79	598.55	13.20	10.45	16.45				

Post hoc analysis used cervical swabs only to ascertain end points; all self-collected swabs after month 3 were excluded. Methods are otherwise the same as described in Table 2. No multiplicity adjustments were performed.

The absolute reduction in the HPV 16/18 mITT cohort for cumulative incident-persistent HPV 16/18 infection was −16.0% (95% CI −19.5 to −12.5%) for the nonavalent group and −15.8% (95% CI −19.3 to −12.3%) for the bivalent group; an absolute incidence of 0.2% (95% CI 0–0.6%) in the nonavalent vaccine group and 0.4% (95% CI 0–1.0 %) in the bivalent group compared to 16.2% (95% CI 12.7–19.7%) in the control group. For the HPV 16/18/31/33/45/52/58 mITT cohort, the absolute reduction in persistent HPV 16/18/31/33/45/52/58 infection was −30.1% (95% CI −36.1 to −24.2%) for the nonavalent vaccine; an absolute incidence of 1.6% (95% CI 0.2–2.9%) in the nonavalent vaccine group compared to 31.7% (95% CI 25.9–37.4%) in the control group.

## Discussion

Three years after vaccine administration, the high efficacy of both single-dose bivalent or nonavalent HPV vaccine was sustained and durable against vaccine-specific oncogenic HPV infection. Protection against type-specific incident-persistent infection was ≥98% for bivalent and nonavalent vaccine protection against HPV 16/18 and >95% for nonavalent vaccine protection against HPV 16/18/31/33/45/52/58, which cause 70% and 90% of cervical cancer cases, respectively. This together with observed high reductions in the absolute cumulative incidence, the potential for public health impact in the context of disparities by HDI in cervical cancer incidence and mortality^1^ is substantial. Saliently, there is high certainty of VE of at least 90% against HPV 16/18; the lower confidence interval limit for the bivalent and nonavalent VE.

Taken in context, these data contribute to a suite of studies that provide evidence for single-dose HPV VE. The Costa Rica vaccine trial (CVT)^12^ provided the first observational data for bivalent single-dose HPV vaccine effectiveness and recently demonstrated durability over 16 years^19^. The DoRIS study demonstrated that a single-dose nonavalent or bivalent HPV vaccine produced robust immune responses similar to two doses and three doses among 9–14-year-old girls^20^. The IARC-India study reported durability of single-dose quadrivalent HPV vaccine effectiveness over a decade^11^. Thus, consistent evidence on the efficacy and durability of single-dose HPV vaccination supports the WHO guidance for single-dose implementation to increase vaccine coverage. In mathematical modeling analyses of scale-up, implementation of routine single-dose immunization has the potential to avert most cervical cancer cases compared to two doses, with a durability of 20–30 years, in low-HDI settings^21^. Further, single-dose vaccination can increase coverage among girls in the 9–14-year-old group before they age out of vaccine eligibility and provide catch-up vaccination for those who may have missed the immunization due to the COVID-19 pandemic or other reasons.

Of the 11.6 million cases of cervical cancer expected globally among girls born between 2005 and 2014, 75% of the burden will be concentrated in 25 countries largely in Africa and Asia, highlighting the need to focus prevention efforts among recently born girls^5^. Overall, the rate of incident persistent HPV infection in this population of African adolescent girls and young women was high; 13.8 per 100 woman-years in the control group, underscoring the need for effective, scalable vaccine programs that can achieve high coverage and reduce this high incidence of HPV infection and ultimately cervical cancer^22,23^. Catch-up vaccination programs for adolescents and young people aged 15–20 years, who do not qualify for current vaccination programs, have the potential to avert oncogenic persistent infections. Through this head-to-head comparison of bivalent and nonavalent HPV vaccines, sustained VE was demonstrated in the context of high HPV prevalence. Single-dose HPV vaccination could increase vaccine access and coverage and offer a cost-effective strategy for cervical cancer prevention.

The vaccines’ underlying immunological mechanism of action could explain the observed VE. The vaccines contain monomers that self-assemble into capsomers and VLPs, a highly immunogenic structure mimicking the ordered, repetitive virus epitope structure and allowing for crosslinking of B cell receptors^8^. This induces high levels of virIon-neutralizing serum antibodies and long-lasting plasma cells, supporting effective and durable VE. We did not see evidence of cross-protection with a single dose of the bivalent vaccine and it may be that two doses are required for cross-protection for the closely related HPV 31/33/45. The confidence interval did not include previous estimates of the multi-dose bivalent strategy of 50% cross-protection for HPV 31/33/45 (ref. ^5^).

The study has several strengths, including its randomized, double-blind controlled design, high retention rate, use of cervical HPV DNA as the outcome measure, determination of incident persistent HPV DNA, head-to-head comparison of the licensed bivalent and nonavalent HPV vaccines in protection against persistent infection with oncogenic HPV types, and duration of follow-up. Moreover, the trial successfully enrolled individuals exposed to HPV infection and retained them in all randomized groups, facilitating a rapid evaluation of single-dose efficacy. Compared to the 18-month analysis, the final analysis VE estimates are stable through 36 months, with higher point-estimates and tighter confidence intervals, as additional follow-up time was accrued beyond the 6–12-month buffer period, during which infections that were prevalent at baseline but not detected at follow-up, and may contribute to a lower estimate of VE^13,14^.

We acknowledge that the study has limitations. First, the median duration of follow-up is 35 months and longer-term durability of single-dose VE in a randomized trial would strengthen the evidence as HPV exposure continues through adulthood. Observational data for single-dose HPV vaccination support efficacy over a decade^11^. While participants in the control group have received single-dose HPV vaccination, we are collecting additional data in this cohort to 54 months post-vaccination^24^. The antibody plateau level for single-dose HPV vaccination is reached by 12 months^9^, suggesting that we have observed steady state efficacy. Second, 7% of primary end-point swabs were self-collected and 93% were provider-collected. All swabs would ideally be collected with one modality; however, the correlation between self-collected vaginal and provider-collected cervical swabs is high^25^ and there was no difference in the results when self-collected swabs were excluded. For the preplanned subgroup analyses by sexually transmitted infection (STI) status, the subgroups were defined at enrollment and may have changed over time; however, the incidence of persistent non-vaccine HPV types was comparable through the study in the three study groups. Finally, while the GST-ELISA multiplex assay used to exclude participants with HPV antibodies at enrollment demonstrated overall agreement of 89% with the gold standard secreted alkaline phosphatase pseudovirion-based neutralization assay^26^, misclassification of participants as antibody naive would not be different by study group. Further in sensitivity analysis including participants with HPV antibodies at baseline, overall VE was in keeping with the primary findings (Table 2a,b).

Globally cervical cancer is a leading cause of morbidity and mortality among women in mid-life; it is the second most common cancer and the greatest contributor to cancer-related mortality among women in southern and East Africa carrying a high cost to women, their families and communities^1,27^. Focusing on the cohort of girls and adolescent women who are at risk of developing cervical cancer if not vaccinated, global HPV 16/18 vaccination of women born between 2005 and 2014 would avert 8 million (7.8–8.3) cervical cancer cases and HPV 16/18/31/33/45/52/58 vaccination would avert 10.2 million (10.0–10.6) cases, with 70% of cases averted in low-to-middle HDI countries^6^. Cervical cancer is almost entirely preventable through HPV vaccination. Single-dose HPV vaccination could serve to close the gap between the WHO goal of 90% HPV vaccination coverage by 2030 and the 15% of girls globally currently vaccinated^7^, alleviate vaccine supply constraints^27^ and provide global policymakers with options to optimally allocate existing HPV vaccine supply. The most recent Cochrane review of the efficacy of single-dose HPV vaccination highlighted that there was moderate evidence on the durability of VE, which we have now provided with robust data over 3 years^13^. Single-dose HPV vaccination could facilitate rapid scale-up of vaccination worldwide.

Over 36 months, single-dose HPV vaccination offered high protection, >95% VE in preventing incident, persistent HPV 16/18/31/33/45/52/58 infection, with the lower bound of the confidence interval at almost 90% (89%) indicating a high minimum level of efficacy. Single-dose HPV vaccination was safe with no vaccine-related SAEs. These data add to the growing suite of evidence to support single-dose HPV vaccination implementation.

## Methods

### Study design

This randomized, multicenter, double-blind, parallel, three-group controlled, superiority trial tested the efficacy of single-dose bivalent (HPV 16/18) and single-dose nonavalent (HPV 16/18/31/33/45/52/58/6/11) HPV vaccination, as described in the published protocol paper^17^ and in the report of the primary results^10^. The study was conducted at three Kenya Medical Research Institute (KEMRI) clinical sites in Kisumu, Thika and Nairobi.

In GAVI-funded countries, including Kenya, multi-dose HPV vaccination is offered to 9–14-year-old girls through the national immunization program. Catch-up vaccination for adolescent girls and young women 15 years of age and older is not provided. Cervical cancer screening is offered to older women instead. We conducted a clinical trial to test the efficacy of single-dose HPV vaccination among young women aged 15-20 years within the context of cytological screening for dysplastic lesions. This was determined to be ethical, as vaccination for this age group in Kenya and many low-HDI countries is not currently supported through national programs or global immunization bodies^18^.

### Participants

Participants were eligible for the study if they were born female, aged 15–20 years old inclusive, were sexually active with one to five sexual partners reported in their lifetime, and planned to reside in the study area for 37 months. The exclusion criteria were people living with HIV for whom few data on single-dose HPV VE are available, history of previous HPV vaccination, allergies to vaccine components of latex, pregnancy, hysterectomy, history of autoimmune, degenerative or genetic diseases, and investigator discretion regarding participant safety. Sex assigned at birth was assessed through participant self-report at screening. Participants were recruited through community outreach. All participants, and their parents/guardians in the case of minors, provided written informed consent, which included counseling about randomization, risks and benefits of participation, study procedures and their rights as research participants.

### Randomization and masking

Meningococcal vaccination was chosen as the control because meningococcal antibodies offer potential clinical benefits and do not impact HPV outcomes. Participants were randomized to (1) nonavalent HPV vaccination (Gardasil-9), (2) bivalent HPV vaccination (Cervarix) or (3) meningococcal (control) vaccination. Following randomization, a single dose of each vaccine was administered.

An unblinded statistical analyst generated the randomization sequence using SAS v.9.4. Randomization was stratified by site, using a fixed block size of 15 and a 1:1:1 allocation. Blinded study assignment was implemented via www.randomize.net. Study staff, participants, investigators, clinic staff, laboratory technicians, the end points adjudication committee members and other study team members did not have access to the randomization codes, except for the unblinded statistical analysts and unblinded pharmacists at each site. At the conclusion of the enrollment visit, an unblinded pharmacist entered the participant identification number (PTID) on randomize.net, obtained the next sequential intervention assignment, recorded the PTID and randomization identifier on an eCRF, drew up the vaccine in a masked syringe and administered the vaccination via the intramuscular route. An independent observer, not on the study team, observed the masked vaccination to assess the success of masking.

### Procedures

Potential participants completed eligibility screening with a provider, including a detailed medical history, collection of external genital (labial/vulvar/perineal), lateral vaginal, and cervical swabs for HPV DNA testing, and serum for HPV antibody testing. Participants received cytological screening for cervical cancer screening at enrollment. Sexual and reproductive health services (contraception, STI diagnosis and treatment, HIV testing and HIV pre-exposure prophylaxis) were offered at enrollment and every visit. Participants also received counseling including services for mental health. All questionnaires used electronic case report forms (eCRFs) (DFexplore Software, DF/Net Research).

Participants had study visits at months 3, 6 and then every 6 months for up to 36 months. Providers administered clinical questionnaires and collected a cervical swab at each 6-month visit. Participants self-collected vaginal swabs using validated instructions at month 3; self-collected swabs, which have similar accuracy compared to provider-collected cervical swabs^25^, were available at subsequent follow-up visits by participant choice or to comply with COVID-19 research restrictions.

Following dissemination and WHO review of the month 18 primary results, participants were offered vaccination at their next study visit, which was at either month 30 or 36, so as not to delay vaccine receipt. Participants provided a final analysis cervical swab before vaccination. Participants in the meningococcal group received the nonavalent HPV vaccine and those in the HPV vaccine groups received the meningococcal vaccine.

### Laboratory methods

HPV DNA genotyping was conducted using the Anyplex II HPV28 assay (Seegene), a multiplexed type-specific real-time PCR-based assay that detects 28 HPV types^28,29^ at the University of Washington (UW) East Africa STI Laboratory, Mombasa, Kenya with standard proficiency testing^30^. For HPV-positive samples, a low (+), intermediate (++), or high (+++) positivity was indicated; + or greater was considered positive. The assay runs included negative and positive controls and the housekeeping human gene, β-globin, as an internal control. Runs were performed with CFX96 Real-time PCR System (Bio-Rad).

Serum specimens were shipped to the UW and tested at the Galloway Laboratory, Fred Hutchinson Cancer Research Center. HPV IgG antibodies were detected using a multiplex Luminex assay^31,32^. The mean pre-established fluorescent intensity seropositivity cutoffs for HPV 16/18/31/33/45/52/58 were used^10^.

Sexually transmitted infections (*N.* *gonorrhoeae, C.* *trachomatis* or *T.* *vaginalis*) were assessed by nucleic acid amplification testing (APTIMA; Hologic/GenProbe) at the UW East Africa STI Laboratory; HSV-2 was evaluated by the Focus ELISA and BV was evaluated using the Nugent score at the National Quality Control Laboratory, Nairobi, Kenya.

### Outcomes

The primary trial end point was incident-persistent cervical vaccine type-specific HPV infection among participants who were vaccine-type HPV naive at enrollment. Persistent HPV infection, a surrogate marker for cervical dysplasia/precancer, was defined as high-risk vaccine-type-specific HPV (HPV 16/18 for the bivalent vaccine and HPV 16/18/31/33/45/52/58 for the nonavalent vaccine) detected at two consecutive visits after the month 3 visit, which were obtained no less than 4 months apart (with the same HPV type at both time points). Cervical swabs were tested for the primary end point; vaginal swabs were substituted if necessary. This analysis included follow-up through month 36 to evaluate the durability of VE.

Secondary analyses assessed VE in the sensitivity cohorts and subgroup analyses. The prespecified subgroups were the presence of co-infections (chlamydia, gonorrhea, HSV-2, trichomoniasis, syphilis and BV), self-reported condom use, number of self-reported lifetime sexual partners (1 versus 2+) and contraception method use.

Safety was assessed through adverse event reporting following Division of Allergy and Infectious Diseases Guidelines^33^. Participants were monitored for adverse events 30 min after vaccination, asked about adverse events at each study visit and reported adverse events outside of study visits. The study clinical monitor followed SAEs, including permission to access medical records. SAEs were recorded on a CRF. The study Principal Investigator and clinical monitor determined the relatedness of SAEs to vaccination. We followed the KEMRI Scientific Ethics Review Unit’s (SERU’s) guidance for reporting SAEs.

### Statistical analysis

The study was powered for the 18-month analysis, which included follow-up through 18 months and has been previously published^10^. The sample size calculations assumed a combined persistent HPV 16/18/31/33/45/52/58 annual incidence of 5%, single-dose VE of 75%, and loss-to-follow-up of 10% with a fixed follow-up time of 12 months. Sample size calculations assumed that 52% of participants would meet the requirements for inclusion in the 18-month analysis based on the observed prevalence of HPV infection in similar settings^34^. Assuming a proportional hazards model (seqDesign in R) with 80% power to detect 75% efficacy, a sample size of 2,250 participants was planned.

We used Cox proportional hazards models stratified by study site to estimate the hazard ratios (HRs) of the interventions versus control for the primary and sensitivity analyses. Models for the sensitivity analyses used crude incidence rate ratios instead of the Cox model when no events were observed in a group. Follow-up was calculated as days since the month 3 visit for the primary analysis and days since month 6 for the extended-sensitivity analysis until the first persistent infection. Participants who did not reach this outcome were censored at the last study visit with HPV testing where they did not meet the criteria for persistent infection. VE was expressed as 1 − HR (or relative risk). The log-rank test stratified by study site was used to calculate the *P* value for each comparison (one degree of freedom). Cumulative incidence curves of time to infection were calculated by intervention group using Kaplan–Meier methods. Efficacy analyses were performed on the month 36 mITT cohorts. In post hoc analysis, we evaluated the absolute difference in cumulative incidence of HPV from the Kaplan–Meier survival estimates at month 36. We calculated the rates of chlamydia and gonorrhea during follow-up by assigned group.

Participants included in the primary analysis tested HPV DNA negative (external genital/lateral vaginal, and cervical swabs) at enrollment and at month 3, by self-collected vaginal swab and HPV antibody negative at enrollment in the mITT cohort. The ITT included all randomized participants. For inclusion in the HPV 16/18 mITT cohort, participants were HPV 16/18 naive. Similarly, for the HPV 16/18/31/33/45/52/58 mITT cohort, participants were HPV 16/18/31/33/45/52/58 naive. Participants without swabs after month 3 did not contribute follow-up time in the primary analysis. Participants in the bivalent vaccine group were excluded from the HPV 16/18/31/33/45/52/58 analysis as the study was not powered to detect cross-protection. Participants who seroconverted to HIV during follow-up are included in analyses.

Sensitivity analyses were planned for the following subsets: participants who tested HPV DNA negative at enrollment and month 3, regardless of antibody status at enrollment (sensitivity cohort) and participants who tested HPV DNA negative at enrollment, month 3, and month 6 and antibody negative at enrollment (extended-sensitivity cohort); the sensitivity cohort was a less conservative definition of an HPV-naive cohort and the extended-sensitivity cohort more closely matched the analysis cohort for HPV vaccine licensure trials. The extended-sensitivity cohort excluded participants who might have had prevalent HPV infection at enrollment that was not detected. Safety was assessed among all participants; the three groups were compared using Fisher’s exact test. Exploratory analysis evaluated cross-protection of the bivalent vaccine against HPV 31/33/45. We performed all analyses using SAS software, v.9.4 (SAS Institute) and R (v.4.2.2).

An independent Data Safety and Monitoring Board was constituted to review study progress, participant safety and the primary outcome, and met annually. The trial is registered at ClinicalTrials.gov (NCT03675256).

### Ethics and inclusion statement

Data for this study, including from Kenya, were collected via eCRFs in Kenya. Seven colleagues (M.A.O., E.A.B., B.N., I.W., C.B., S.K. and N.R.M.), including the senior author (N.R.M.) are from Kenya, a low-and-middle-income country and one other (R.V.B.) is South African and is now based in a high-income country. We fully endorse and are committed to the Nature Portfolio journals’ guidance on low-and-middle-income country authorship and inclusion.

This research is locally relevant to Kenya and other countries that have not achieved the 90% HPV vaccine coverage goal.

The KEMRI SERU (nos. 3745 and 3741) and the Massachusetts General Hospital Institutional Review Board (no. 2022P001178) approved the study. Study participation may have carried stigmatization associated with vaccination. The data collection and analysis techniques employed raised no risks pertaining to incrimination, discrimination, animal welfare, the environment, health, safety, security or other personal risks. All HPV and STI testing was conducted at local laboratories. Serum for specialized HPV antibody testing was shipped to Seattle for testing. No cultural artifacts or associated traditional knowledge has been transferred out of any country. In preparing the manuscript, the authors reviewed relevant studies from Kenya.

### Reporting summary

Further information on research design is available in the Nature Portfolio Reporting Summary linked to this article.

## Online content

Any methods, additional references, Nature Portfolio reporting summaries, source data, extended data, supplementary information, acknowledgements, peer review information; details of author contributions and competing interests; and statements of data and code availability are available at 10.1038/s41591-023-02658-0.

## Supplementary information

Supplementary Information Supplementary Tables 1 and 2

Reporting Summary

## Figures and Tables

**Extended Data Table. 1:**
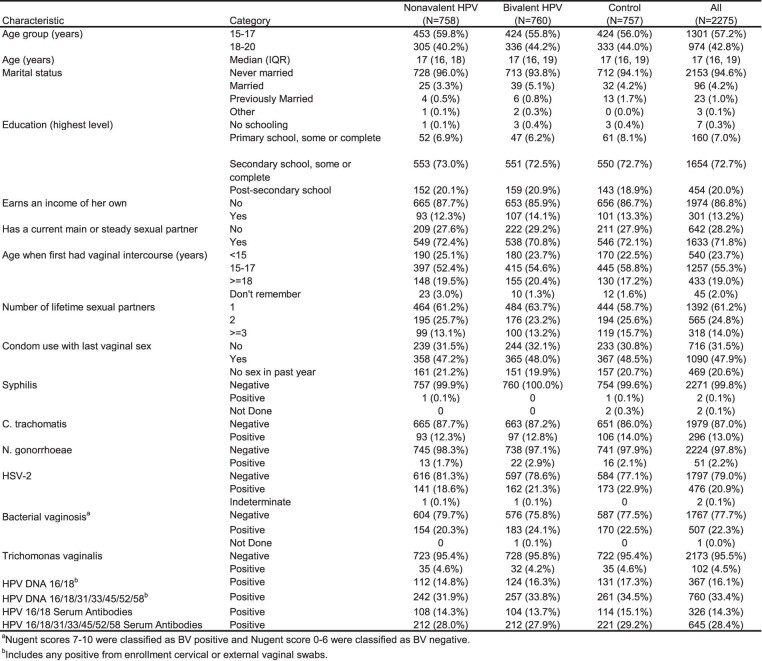
Baseline characteristics of the ITT population

**Extended Data Table. 2:**
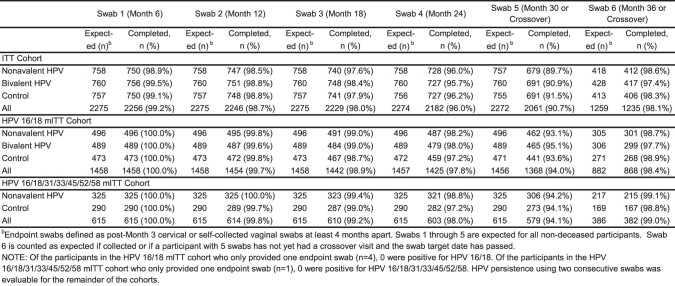
Completeness of end point swab collection

**Extended Data Table. 3:**
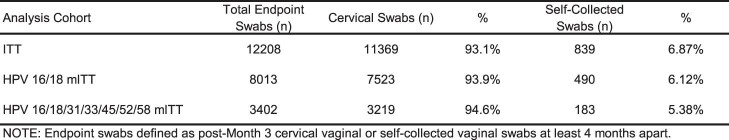
Summary of tested end point swabs and swab collection types

**Extended Data Table. 4:**
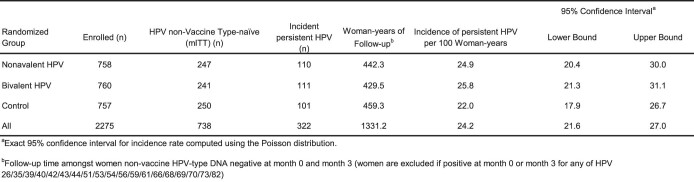
Exploratory analysis: incidence of persistent HPV types (combined non-vaccine types, mITT sensitivity cohort)

**Extended Data Table. 5:**
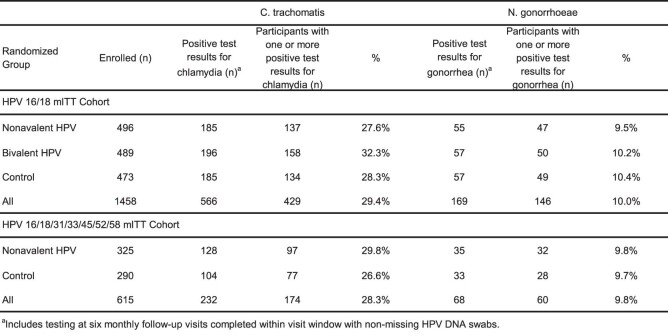
Summary of follow-up laboratory results by randomized group (mITT cohorts)

**Extended Data Table. 6:**
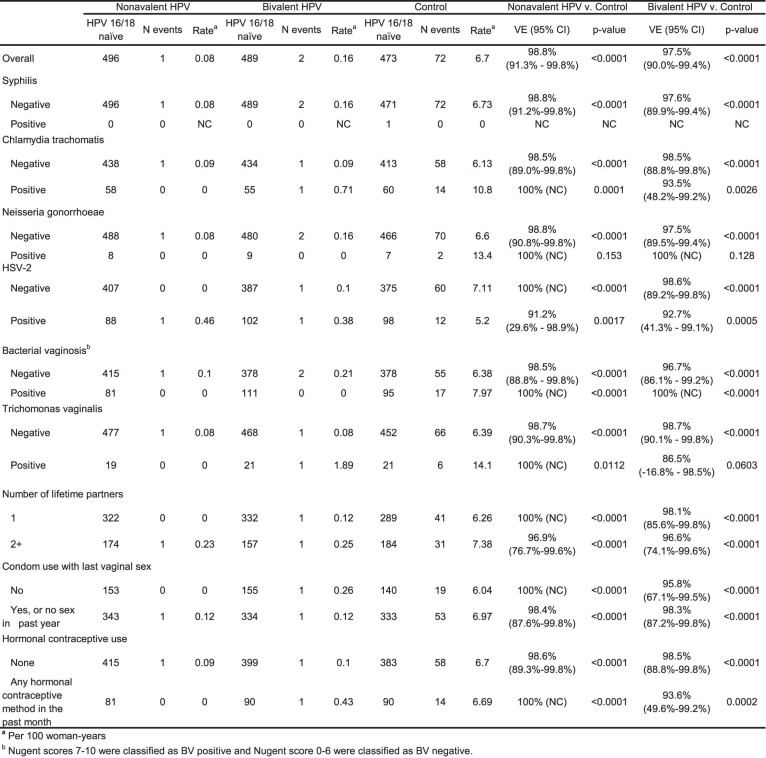
Incidence of persistent HPV 16/18 and vaccine efficacy overall and within subgroups defined by lab results or self-reported behavior at enrollment (mITT cohort)

Subgroups are defined by lab results or self-reported behavior at the enrollment visit. No adjustments for multiplicity were done. Two-sided log-rank p-values are computed for each comparison using the log-rank test.

**Extended Data Table. 7:**
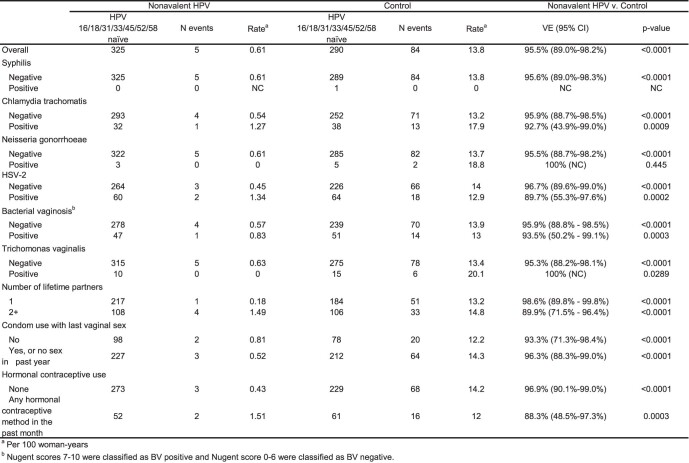
Incidence of persistent HPV 16/18/31/33/45/52/58 and vaccine efficacy overall and within subgroups defined by lab results or self-reported behavior at enrollment (mITT cohort)

Subgroups are defined by lab results or self-reported behavior at the enrollment visit. No adjustments for multiplicity were done. Two-sided log-rank p-values are computed for each comparison using the log-rank test.

**Extended Data Table. 8:**
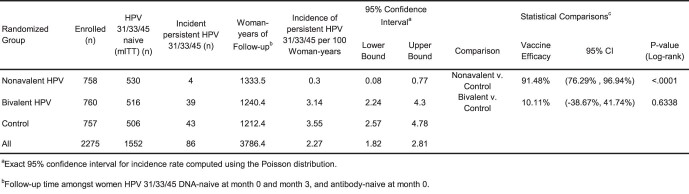
Exploratory analysis: Incidence of persistent HPV 31/33/45 (HPV 31/33/45 mITT cohort)

Two-sided log-rank p-values are computed for each comparison using the log-rank test. No adjustments for multiplicity were done.

## Data Availability

Data are available subject to controlled access because additional analysis requires regulatory approval. This study was conducted with approval from the KEMRI SERU, which requires that data from studies (including de-identified data) are released only after SERU has provided written approval for additional analyses. To request these data and facilitate submission to SERU for additional analyses, please contact the KEN SHE Scientific Committee at lnakatsuka@partners.org. A complete de-identified dataset and code book sufficient to reproduce the study findings will be made available 1 year after study closeout upon written request after approval from SERU.

## Code availability

The custom code and code book sufficient to reproduce the study findings will be made available 1 year after study closeout upon written request after approval from the KEN SHE Scientific Committee. Please contact the KEN SHE Scientific Committee at lnakatsuka@partners.org.

## References

[1] Singh, D. et al. Global estimates of incidence and mortality of cervical cancer in 2020: a baseline analysis of the WHO Global Cervical Cancer Elimination Initiative. *Lancet Glob. Health* **11**, e197–e206 (2023). 36528031 10.1016/S2214-109X(22)00501-0 PMC9848409

[2] Harper, D. M. & DeMars, L. R. HPV vaccines - a review of the first decade. *Gynecol. Oncol.* **146**, 196–204 (2017). 28442134 10.1016/j.ygyno.2017.04.004

[3] Huh, W. K. et al. Final efficacy, immunogenicity, and safety analyses of a nine-valent human papillomavirus vaccine in women aged 16-26 years: a randomised, double-blind trial. *Lancet* **390**, 2143–2159 (2017). 28886907 10.1016/S0140-6736(17)31821-4

[4] World Health Organization. *A Global Strategy for Elimination of* Cervical Cancer www.who.int/activities/a-global-strategy-for-elimination-of-cervical-cancer (2020).

[5] Drolet, M., Benard, E., Perez, N. & Brisson, M., H. P. V. Vaccination Impact Study Group. Population-level impact and herd effects following the introduction of human papillomavirus vaccination programmes: updated systematic review and meta-analysis. *Lancet* **394**, 497–509 (2019). 31255301 10.1016/S0140-6736(19)30298-3 PMC7316527

[6] Bonjour, M. et al. Global estimates of expected and preventable cervical cancers among girls born between 2005 and 2014: a birth cohort analysis. *Lancet Public Health* **6**, e510–e521 (2021). 33864738 10.1016/S2468-2667(21)00046-3 PMC8225515

[7] Bruni, L. et al. HPV vaccination introduction worldwide and WHO and UNICEF estimates of national HPV immunization coverage 2010-2019. *Prevent. Med.* **144**, 106399 (2021). 10.1016/j.ypmed.2020.106399 33388322

[8] Lowy, D. R. HPV vaccination to prevent cervical cancer and other HPV-associated disease: from basic science to effective interventions. *J. Clin. Invest.* **126**, 5–11 (2016). 26727228 10.1172/JCI85446 PMC4701560

[9] Baisley, K. et al. Comparing one dose of HPV vaccine in girls aged 9-14 years in Tanzania (DoRIS) with one dose of HPV vaccine in historical cohorts: an immunobridging analysis of a randomised controlled trial. *Lancet Glob. Health* **10**, e1485–e1493 (2022). 36113532 10.1016/S2214-109X(22)00306-0 PMC9638025

[10] Barnabas, R. V. et al. Efficacy of single-dose HPV vaccination among young African women. *NEJM Evid.* **1**, EVIDoa2100056 (2022). 35693874 10.1056/EVIDoa2100056 PMC9172784

[11] Basu, P. et al. Vaccine efficacy against persistent human papillomavirus (HPV) 16/18 infection at 10 years after one, two, and three doses of quadrivalent HPV vaccine in girls in India: a multicentre, prospective, cohort study. *Lancet Oncol.*10.1016/S1470-2045(21)00453-8 (2021). 10.1016/S1470-2045(21)00453-8 PMC8560643 34634254

[12] Kreimer, A. R. et al. Evidence for single-dose protection by the bivalent HPV vaccine: review of the Costa Rica HPV vaccine trial and future research studies. *Vaccine* **36**, 4774–4782 (2018). 29366703 10.1016/j.vaccine.2017.12.078 PMC6054558

[13] Henschke, N. et al. *Efficacy, Effectiveness and Immunogenicity of One Dose of HPV Vaccine Compared with No Vaccination, Two Doses, or Three Doses* (Cochrane Response, 2022).

[14] Markowitz, L. E. et al. Human papillomavirus vaccine effectiveness by number of doses: updated systematic review of data from national immunization programs. *Vaccine* **40**, 5413–5432 (2022). 35965239 10.1016/j.vaccine.2022.06.065 PMC9768820

[15] Whitworth, H. S. et al. Efficacy and immunogenicity of a single dose of human papillomavirus vaccine compared to no vaccination or standard three and two-dose vaccination regimens: a systematic review of evidence from clinical trials. *Vaccine* **38**, 1302–1314 (2020). 31870572 10.1016/j.vaccine.2019.12.017

[16] Umutesi, G. et al. HPV vaccination in Kenya: a study protocol to assess stakeholders’ perspectives on implementation drivers of HPV vaccination and the acceptability of the reduced dose strategy among providers. *Front. Health Serv.* **3**, 1233923 (2023). 37600926 10.3389/frhs.2023.1233923 PMC10433907

[17] Barnabas, R. V. et al. Single-dose HPV vaccination efficacy among adolescent girls and young women in Kenya (the KEN SHE study): study protocol for a randomized controlled trial. *Trials* **22**, 661 (2021). 34579786 10.1186/s13063-021-05608-8 PMC8475401

[18] Shadab, R. et al. Key ethical considerations to guide the adjudication of a single-dose HPV vaccine schedule. *Hum. Vaccines Immunother.*10.1080/21645515.2021.1917231 (2021). 10.1080/21645515.2021.1917231 PMC8920253 34010096

[19] Romero, B. et al. Durability of HPV 16-18 antibodies 16 years after a single dose of the bivalent HPV vaccine: the Costa Rica HPV vaccine trial. in *IPVC 2023* (IPVC, 2023).

[20] Watson-Jones, D. et al. Immunogenicity and safety of one-dose human papillomavirus vaccine compared with two or three doses in Tanzanian girls (DoRIS): an open-label, randomised, non-inferiority trial. *Lancet Glob. Health* **10**, e1473–e1484 (2022). 36113531 10.1016/S2214-109X(22)00309-6 PMC9638030

[21] Benard, E. et al. Potential population-level effectiveness of one-dose HPV vaccination in low-income and middle-income countries: a mathematical modelling analysis. *Lancet Public Health* **8**, e788–e799 (2023). 37777288 10.1016/S2468-2667(23)00180-9 PMC10557953

[22] Harper, D. M. et al. Efficacy of a bivalent L1 virus-like particle vaccine in prevention of infection with human papillomavirus types 16 and 18 in young women: a randomised controlled trial. *Lancet* **364**, 1757–1765 (2004). 15541448 10.1016/S0140-6736(04)17398-4

[23] Joura, E. A. et al. A 9-valent HPV vaccine against infection and intraepithelial neoplasia in women. *New Engl. J. Med.* **372**, 711–723 (2015). 25693011 10.1056/NEJMoa1405044

[24] Follmann, D. et al. A deferred-vaccination design to assess durability of COVID-19 vaccine effect after the placebo group is vaccinated. *Ann. Intern. Med.* **174**, 1118–1125 (2021). 33844575 10.7326/M20-8149 PMC8099035

[25] Polman, N. J. et al. Performance of human papillomavirus testing on self-collected versus clinician-collected samples for the detection of cervical intraepithelial neoplasia of grade 2 or worse: a randomised, paired screen-positive, non-inferiority trial. *Lancet Oncol.* **20**, 229–238 (2019). 30658933 10.1016/S1470-2045(18)30763-0

[26] Robbins, H. A. et al. Glutathione-*S*-transferase L1 multiplex serology as a measure of cumulative infection with human papillomavirus. *BMC Infect. Dis.* **14**, 120 (2014). 24588945 10.1186/1471-2334-14-120 PMC3973893

[27] Prem, K. et al. Global impact and cost-effectiveness of one-dose versus two-dose human papillomavirus vaccination schedules: a comparative modelling analysis. *BMC Med.*10.1186/s12916-023-02988-3 (2023). 10.1186/s12916-023-02988-3 PMC10463590 37635227

[28] Jung, S., Lee, B., Lee, K. N., Kim, Y. & Oh, E. J. Clinical validation of Anyplex II HPV HR detection test for cervical cancer screening in Korea. *Arch. Pathol. Lab. Med.* **140**, 276–280 (2016). 26927723 10.5858/arpa.2015-0117-OA

[29] Hesselink, A. T. et al. Clinical validation of the HPV-risk assay, a novel real-time PCR assay for detection of high-risk human papillomavirus DNA by targeting the E7 region. *J. Clin. Microbiol.* **52**, 890–896 (2014). 24391196 10.1128/JCM.03195-13 PMC3957775

[30] Eklund, C., Forslund, O., Wallin, K. L. & Dillner, J. Continuing global improvement in human papillomavirus DNA genotyping services: the 2013 and 2014 HPV LabNet international proficiency studies. *J. Clin. Virol.* **101**, 74–85 (2018). 29433017 10.1016/j.jcv.2018.01.016

[31] Rowhani-Rahbar, A. et al. Antibody responses in oral fluid after administration of prophylactic human papillomavirus vaccines. *J. Infect. Dis.* **200**, 1452–1455 (2009). 19698077 10.1086/606026 PMC3392559

[32] Waterboer, T. et al. Multiplex human papillomavirus serology based on in situ-purified glutathione s-transferase fusion proteins. *Clin. Chem.* **51**, 1845–1853 (2005). 16099939 10.1373/clinchem.2005.052381

[33] RSC. *DAIDS Adverse Event Grading Tables* rsc.niaid.nih.gov/clinical-research-sites/daids-adverse-event-grading-tables (2018).

[34] Watson-Jones, D. et al. High prevalence and incidence of human papillomavirus in a cohort of healthy young African female subjects. *Sex. Transm. Infect.* **89**, 358–365 (2013). 23486859 10.1136/sextrans-2012-050685 PMC3717757

