# Supplementary material for: Durability of single-dose HPV vaccination in young Kenyan women: randomized controlled trial 3-year results
Source: Nat Med. 2023 Dec 4;29(12):3224–32. doi: 10.1038/s41591-023-02658-0 (PMC10719107; doi:10.1038/s41591-023-02658-0)
Supplement: Supplementary file 1 — Supplementary Tables 1 and 2 [file 41591_2023_2658_MOESM1_ESM.pdf]

nature medicine

Article

https://doi.org/10.1038/s41591-023-02658-0

Durability of single-dose HPV vaccination in young Kenyan women: randomized controlled trial 3-year results

In the format provided by the authors and unedited

Table of Contents

Supplementary Tables 2

Supplementary Table 1 | Summary of mITT endpoint HPV types by randomized group (mITT Cohorts) 2

Supplementary Table 2 | Incidence of persistent HPV (vaccine types) by month 36 (HPV type-specific mITT cohorts) 3

Supplementary Tables

Supplementary Table 1 | Summary of mITT endpoint HPV types by randomized group (mITT Cohorts)

	HPV 16/18 mITT				HPV 16/18/31/33/45/52/58 mITT		
**HPV Type[a]**	**Nonavalent HPV (n)**	**Bivalent HPV (n)**	**Control (n)**	**Total (n)**	**Nonavalent HPV (n)**	**Control (n)**	**Total (n)**
16	1	2	50	53	1	20	21
16,18	0	0	1	1	0	0	0
18	0	0	21	21	0	10	10
16,52	n/a	n/a	n/a	n/a	0	1	1
16,52,58	n/a	n/a	n/a	n/a	0	1	1
16,58	n/a	n/a	n/a	n/a	0	1	1
18,58	n/a	n/a	n/a	n/a	0	1	1
31	n/a	n/a	n/a	n/a	1	5	6
31,58	n/a	n/a	n/a	n/a	0	1	1
33	n/a	n/a	n/a	n/a	0	2	2
33,52	n/a	n/a	n/a	n/a	0	1	1
45	n/a	n/a	n/a	n/a	0	9	9
45,52	n/a	n/a	n/a	n/a	0	2	2
52	n/a	n/a	n/a	n/a	0	13	13
52,58	n/a	n/a	n/a	n/a	0	1	1
58	n/a	n/a	n/a	n/a	3	16	19
Total	1	2	72	75	5	84	89

[a]Vaccine-specific HPV type(s) detected at two consecutive visits at least four months apart. If two or more HPV types are listed, both/all types were detected at the consecutive visits.
n/a – Not applicable for this analysis cohort.

Supplementary Table 2 | Incidence of persistent HPV (vaccine types) by month 36 (HPV type-specific mITT cohorts)

HPV Type	Randomized Group	Enrolled (n)	HPV type-naive (mITT) (n)	Incident persistent HPV (n)	Woman-years of Follow-up[b]	Incidence of persistent HPV per 100 Woman-years	95% Confidence Interval[a] Lower Bound	Upper Bound
16	Nonavalent HPV	758	521	1	1321.33	0.08	0.00	0.42
	Bivalent HPV	760	528	2	1336.56	0.15	0.02	0.54
	Control	757	503	57	1175.50	4.85	3.67	6.28
	All	2275	1552	60	3833.39	1.57	1.19	2.02
18	Nonavalent HPV	758	652	0	1641.51	0.00	0.00	0.23
	Bivalent HPV	760	635	1	1593.23	0.06	0.00	0.35
	Control	757	623	32	1511.44	2.12	1.45	2.99
	All	2275	1910	33	4746.17	0.70	0.48	0.98
31	Nonavalent HPV	758	622	1	1561.58	0.06	0.00	0.36
	Bivalent HPV	760	619	23	1516.21	1.52	0.96	2.28
	Control	757	596	23	1468.23	1.57	0.99	2.35
	All	2275	1837	47	4546.02	1.03	0.76	1.38
33	Nonavalent HPV	758	695	0	1748.13	0.00	0.00	0.21
	Bivalent HPV	760	691	5	1724.97	0.29	0.09	0.68
	Control	757	680	8	1694.57	0.47	0.20	0.93
	All	2275	2066	13	5167.67	0.25	0.13	0.43
45	Nonavalent HPV	758	605	3	1529.00	0.20	0.04	0.57
	Bivalent HPV	760	600	19	1489.66	1.28	0.77	1.99
	Control	757	597	22	1473.85	1.49	0.94	2.26
	All	2275	1802	44	4492.52	0.98	0.71	1.32
52	Nonavalent HPV	758	590	3	1484.49	0.20	0.04	0.59
	Bivalent HPV	760	587	67	1373.24	4.88	3.78	6.20
	Control	757	558	58	1313.20	4.42	3.35	5.71
	All	2275	1735	128	4170.93	3.07	2.56	3.65
58	Nonavalent HPV	758	573	9	1434.59	0.63	0.29	1.19
	Bivalent HPV	760	569	56	1341.89	4.17	3.15	5.42
	Control	757	564	55	1344.99	4.09	3.08	5.32
	All	2275	1706	120	4121.48	2.91	2.41	3.48

[a]Exact 95% confidence interval for incidence rate computed using the Poisson distribution.
[b]Follow-up time amongst women HPV-type DNA negative at month 0 and month 3 and HPV-type antibody negative at month 0.
