# Supplementary material for: Durability of single-dose HPV vaccination in young Kenyan women: randomized controlled trial 3-year results
Source: Nat Med. 2023 Dec 4;29(12):3224–32. doi: 10.1038/s41591-023-02658-0 (PMC10719107; doi:10.1038/s41591-023-02658-0)
Supplement: Supplementary file 2 — Reporting Summary [file 41591_2023_2658_MOESM2_ESM.pdf]

nature portfolio

Corresponding author(s): Dr. Ruanne Barnabas

Last updated by author(s): Apr 27, 2023

Reporting Summary

Nature Portfolio wishes to improve the reproducibility of the work that we publish. This form provides structure for consistency and transparency in reporting. For further information on Nature Portfolio policies, see our Editorial Policies and the Editorial Policy Checklist.

Statistics

For all statistical analyses, confirm that the following items are present in the figure legend, table legend, main text, or Methods section.

n/a	Confirmed	
☐	☒	The exact sample size (*n*) for each experimental group/condition, given as a discrete number and unit of measurement
☐	☒	A statement on whether measurements were taken from distinct samples or whether the same sample was measured repeatedly
☐	☒	The statistical test(s) used AND whether they are one- or two-sided<br>*Only common tests should be described solely by name; describe more complex techniques in the Methods section.*
☐	☒	A description of all covariates tested
☐	☒	A description of any assumptions or corrections, such as tests of normality and adjustment for multiple comparisons
☐	☒	A full description of the statistical parameters including central tendency (e.g. means) or other basic estimates (e.g. regression coefficient) AND variation (e.g. standard deviation) or associated estimates of uncertainty (e.g. confidence intervals)
☐	☒	For null hypothesis testing, the test statistic (e.g. *F*, *t*, *r*) with confidence intervals, effect sizes, degrees of freedom and *P* value noted<br>*Give P values as exact values whenever suitable.*
☒	☐	For Bayesian analysis, information on the choice of priors and Markov chain Monte Carlo settings
☒	☐	For hierarchical and complex designs, identification of the appropriate level for tests and full reporting of outcomes
☐	☒	Estimates of effect sizes (e.g. Cohen's *d*, Pearson's *r*), indicating how they were calculated

Our web collection on statistics for biologists contains articles on many of the points above.

Software and code

Policy information about availability of computer code

Data collection	Electronic case report forms (eCRFs) in DFExplore (DF/Net Research, Inc. ©, Seattle, WA, US)
Data analysis	SAS software, version 9.4 (SAS Institute, North Carolina, US) and R (version 4.2.2)

For manuscripts utilizing custom algorithms or software that are central to the research but not yet described in published literature, software must be made available to editors and reviewers. We strongly encourage code deposition in a community repository (e.g. GitHub). See the Nature Portfolio guidelines for submitting code & software for further information.

Data

Policy information about availability of data

All manuscripts must include a data availability statement. This statement should provide the following information, where applicable:

- Accession codes, unique identifiers, or web links for publicly available datasets
- A description of any restrictions on data availability
- For clinical datasets or third party data, please ensure that the statement adheres to our policy

Data availability: Data is available subject to controlled access because additional analyses requires regulatory approval. This study was conducted with approval from the Kenya Medical Research Institute (KEMRI) Scientific and Ethics Review Unit (SERU), which requires that data from studies (including de-identified data) are released only after SERU has provided written approval for additional analyses. To request these data and facilitate submission to SERU for additional analyses, please contact the KEN SHE Scientific Committee at lnakatsuka@partners.org.

Code availability: A complete de-identified dataset and code book sufficient to reproduce the study findings will be made available one year after study closeout upon written request after approval from SERU. The custom code and code book sufficient to reproduce the study findings will be made available one year after study closeout upone written request after approval from the KEN SHE Scientific Committee. Please contact the KEN SHE Scientific Committee at lnakatsuka@partners.org.

Research involving human participants, their data, or biological material

Policy information about studies with human participants or human data. See also policy information about sex, gender (identity/presentation), and sexual orientation and race, ethnicity and racism.

Reporting on sex and gender	Self-reported female sex at birth was an inclusion criterion for KEN SHE study enrollment. The KEN SHE study findings pertain to individuals with female sex at birth only.
Reporting on race, ethnicity, or other socially relevant groupings	The KEN SHE study did not collect data on race, ethnicity, or other social groupings. In this manuscript, minimal self-reported socioeconomic data (highest level of schooling and whether a participant earns an income on her own) is used to demonstrate successful randomization and balance of the randomized groups.
Population characteristics	KEN SHE participants are young women, age 15-20, living in proximity to one of the three KEN SHE study sites in Thika, Nairobi, or Kisumu, Kenya. At the time of enrollment, participants had 1 to 5 lifetime sexual partners (inclusive), no history of previous HPV vaccination, and negative pregnancy and HIV rapid test results.
Recruitment	Participants were recruited through community outreach programs, which limits bias associated with facility-based recruitment but this is not a formal general population sample.
Ethics oversight	The Kenya Medical Research Institute (KEMRI) Scientific Ethics Review Unit (SERU) and Massachusetts General Hospital Institutional Review Board (MGH IRB) reviewed and approved the study protocol.

Note that full information on the approval of the study protocol must also be provided in the manuscript.

Field-specific reporting

Please select the one below that is the best fit for your research. If you are not sure, read the appropriate sections before making your selection.

☒ Life sciences ☐ Behavioural & social sciences ☐ Ecological, evolutionary & environmental sciences

For a reference copy of the document with all sections, see nature.com/documents/nr-reporting-summary-flat.pdf

Life sciences study design

All studies must disclose on these points even when the disclosure is negative.

Sample size	The sample size calculations also assumed a combined persistent HPV 16/18/31/33/45/52/58 annual incidence of 5%, single-dose vaccine efficacy of 75%, and loss-to-follow-up of 10% with a fixed follow-up time of 12 months. Sample size calculations assumed that 52% of participants would meet the requirements for inclusion in the primary analysis based on the observed prevalence of HPV infection in similar settings. Assuming a proportional hazards model (seqDesign in R) with 80% power to detect 75% efficacy, a sample size of 2,250 participants was planned.
Data exclusions	As pre-established in the statistical analysis plan, the primary analysis is conducted within the mITT cohorts for HPV 16/18 and HPV 16/18/31/33/45/52/58, which respectively exclude participants DNA- or antibody-positive at enrollment, or DNA-positive at month 3 for the HPV DNA types 16/18 or 16/18/31/33/45/52/58.
Replication	Analysis cohorts (n participants), persistent HPV (n events), incidence rates (n events/100 woman-years), vaccine efficacy (1-HR), and corresponding confidence intervals and p-values were coded independently by two analysts. All findings were replicated.
Randomization	An unblinded statistical analyst generated the randomization sequence using SAS v9.4. Randomization was stratified by site, using a fixed block size of 15 and a 1:1:1 allocation. Blinded study assignment was implemented via http://www.randomize.net (Ottawa, ON, Canada).
Blinding	Study staff, participants, investigators, clinic staff, lab technicians, the endpoints adjudication committee members, and other study team members did not have access to the randomization codes, except for the unblinded statistical analysts and unblinded pharmacists at each site. An unblinded pharmacist entered the participant identification number (PTID) on randomize.net, obtained the next sequential intervention assignment, recorded the PTID and randomization identifier on an eCRF, drew up the vaccine in a masked syringe, and administered the vaccination via the intramuscular route. An independent observer, not on the study team, observed the masked vaccination to assess the success of masking.

Reporting for specific materials, systems and methods

We require information from authors about some types of materials, experimental systems and methods used in many studies. Here, indicate whether each material, system or method listed is relevant to your study. If you are not sure if a list item applies to your research, read the appropriate section before selecting a response.

Materials & experimental systems

n/a	Involved in the study
☒	☐ Antibodies
☒	☐ Eukaryotic cell lines
☒	☐ Palaeontology and archaeology
☒	☐ Animals and other organisms
☐	☒ Clinical data
☒	☐ Dual use research of concern
☒	☐ Plants

Methods

n/a	Involved in the study
☒	☐ ChIP-seq
☒	☐ Flow cytometry
☒	☐ MRI-based neuroimaging

Clinical data

Policy information about clinical studies

All manuscripts should comply with the ICMJE guidelines for publication of clinical research and a completed CONSORT checklist must be included with all submissions.

Clinical trial registration	NCT03675256
Study protocol	The trial protocol paper has been published and the full protocol is available upon request lnakatsuka@partners.org.
Data collection	Study recruitment and enrollment occurred between December 20, 2018 and November 15, 2019, at three study sites in Thika, Nairobi, and Kisumu, Kenya. Data collected between December 20, 2018 and January 9, 2023 are reported in this manuscript. The study was conducted at three Kenya Medical Research Institute (KEMRI) clinical sites in Kisumu, Thika, and Nairobi.
Outcomes	The primary trial endpoint was incident persistent vaccine type-specific HPV infection in cervical and self-collected (when necessary) swabs collected at least four months apart. HPV DNA genotyping was conducted using the Anyplex II HPV28 assay (Seegene, Seoul, South Korea). Secondary analyses assessed vaccine efficacy in the sensitivity cohorts and subgroup analyses. The pre-specified subgroups were the presence of co-infections (chlamydia, gonorrhea, herpes simplex type 2, trichomoniasis, syphilis, and bacterial vaginosis), self-reported condom use, number of self-reported sex partners in the last three months (0-1 vs. 2+), and contraception method use.
